# Limiting postpartum weight retention through early antenatal intervention: the HeLP-her randomised controlled trial

**DOI:** 10.1186/s12966-014-0134-8

**Published:** 2014-10-31

**Authors:** Cheryce L Harrison, Catherine B Lombard, Helena J Teede

**Affiliations:** Monash Centre for Health Research and Implementation (MCHRI), School of Public Health and Preventive Medicine, Monash University, Clayton, Victoria Australia; Diabetes and Vascular Medicine Unit, Monash Health, Clayton, Victoria Australia

**Keywords:** Postpartum weight retention, Gestational weight gain, Pregnancy, Lifestyle intervention, Self-management, Gestational diabetes

## Abstract

**Background:**

Pregnancy is a recognised high risk period for excessive weight gain, contributing to postpartum weight retention and obesity development long-term. We aimed to reduce postpartum weight retention following a low-intensity, self-management intervention integrated with routine antenatal care during pregnancy.

**Methods:**

228 women at increased risk of gestational diabetes, <15 weeks gestation were randomised to intervention (4 self-management sessions) or control (generic health information). Outcomes, collected at baseline and 6 weeks postpartum, included anthropometrics (weight and height), physical activity (pedometer) and questionnaires (health behaviours).

**Results:**

Mean age (32.3 ± 4.7 and 31.7 ± 4.4 years) and body mass index (30.4 ± 5.6 and 30.3 ± 5.9 kg/m^2^) were similar between intervention and control groups, respectively at baseline. By 6 weeks postpartum, weight change in the control group was significantly higher than the intervention group with a between group difference of 1.45 ± 5.1 kg (95% CI: −2.86,-0.02; *p* < 0.05) overall, with a greater difference in weight found in overweight, but not obese women. Intervention group allocation, higher baseline BMI, GDM diagnosis, country of birth and higher age were all independent predictors of lower weight retention at 6 weeks postpartum on multivariable linear regression. Other factors related to weight including physical activity, did not differ between groups.

**Conclusions:**

A low intensity intervention, integrated with standard antenatal care is effective in limiting postpartum weight retention. Implementation research is now required for scale-up to optimise antenatal health care.

**Trial registration:**

Australian New Zealand Clinical Trial Registry Number: ACTRN12608000233325. Registered 7/5/2008.

## Background

Obesity is the fastest growing cause of chronic disease worldwide and its prevalence is progressive, unrelenting and challenging with serious public health and economic implications. Current international trends confirm women are gaining more weight than men, with younger women of reproductive age at highest risk, with rapid weight gain and high levels of obesity [1-3]. Weight related health complications in women are broad, however risk of morbidity and mortality increases even with minor gains in weight (0.5 kg) above a healthy body mass index (BMI) [4].

Increased weight gain and obesity in women is multifactorial, however large cross-sectional [1] and longitudinal [5] studies report weight gain is inversely proportional to current BMI [5] and occurs most rapidly in women aged 18–40, with ~40-50% already overweight or obese [5-7]. Excess weight retention is increasingly common after pregnancy, a recognised high-risk period for weight gain, with 56% of pre-existing overweight and obese women gaining above international Institute of Medicine recommendations for gestational weight gain (GWG) [8]. Excessive GWG impacts negatively on health outcomes during pregnancy [9] and following pregnancy contributes to increased weight retention, a rise in inter-pregnancy weight and is an independent predictor for subsequent development of obesity and related health implications long-term in women [10]. Average weight retained per pregnancy varies by population, however in Australia is ~2-3 kg based on self-reported data [5] with similar or higher findings reported internationally [11] significantly contributing to the background weight gain observed in younger women currently.

Intervening to reduce postpartum weight retention is an important public health initiative; however key gaps remain. Previous studies on systematic review show equivocal findings with small sample sizes noted, difficulties with engagement and ambiguity around ideal setting, delivery and intervention length [12,13]. For these reasons, intervening during pregnancy may be favourable. Pregnancy is recognised as an opportune time with a motivated population, more receptive to lifestyle behaviour change and actively engaged in the healthcare system [14]. Yet, systematic reviews of antenatal interventions highlight gaps, including poor to moderate quality studies with failure to extend follow-up to the postpartum period [15]. Further, results from interventions in higher risk women (i.e. overweight, obese or at risk of GDM) are inconsistent with some [15], but not all [13] systematic reviews reporting reduced excessive GWG following lifestyle intervention. Recent research adds to previous equivocal findings, including the large LIMIT trial reporting no significant effect on GWG in predominantly obese women following moderate-high intensity lifestyle intervention with moderate compliance [16].

We have previously demonstrated the efficacy of a healthy lifestyle program (HeLP-her) for optimising GWG at 28 weeks gestation in women at increased risk of GDM [17]. Here, we aimed to assess sustained impact of the HeLP-her intervention following pregnancy on postpartum weight retention and changes in health behaviours, following early antenatal intervention.

## Methods

### Participants and setting

Detailed study methods have been previously published [17]. In summary, recruitment took place in the antenatal care setting at three large metropolitan tertiary teaching hospitals in metropolitan Melbourne, Victoria, Australia between June 2008 and September 2010 [17]. Women were included if they were ≤15 weeks, had a singleton pregnancy, were overweight (BMI ≥ 25.00 kg/m^2^ or ≥23.00 kg/m^2^ if high risk ethnicity [18]) or obese (BMI ≥ 30.00 kg/m^2^) and at increased risk of GDM as identified by a validated risk prediction tool based on established risk factors including age, BMI, ethnicity and obstetric and family history of diabetes [7]. Exclusion criteria included diagnosed diabetes, a BMI ≥45 kg/m^2^, non-English speaking women or a pre-existing chronic medical condition that prevented full participation or completion of outcome measures (e.g. psychiatric illness, major depression, significant disability).

### Study design

Following initial screening, all eligible women were invited to participate by invitation flyer at their first antenatal booking visit. Those expressing interest were randomly assigned to intervention or control through computer generated randomised sequencing performed by a biostatistician. Allocation concealment was achieved by using sealed opaque envelopes performed by an interventionist. Care providers, investigators and outcome data analysers were blinded to group allocation. Intervention sessions were integrated with routine maternity visits in the clinic setting. All women received standard antenatal care.

### Control and intervention groups

Women allocated to the control group received a brief, single non-interactive education session and brief written resources based on the generic Australian Dietary and Physical Activity Guidelines with no further support provided [19]. Information regarding GWG was not provided.

Women in the intervention group participated in four, 45 minute individual behaviour change lifestyle sessions at 14–16, 20, 24 and 28 weeks gestation. All intervention content was delivered by 28 weeks gestation and underpinned by behavioural principles of the Social Cognitive Theory, informed by our successful lifestyle intervention program (HeLP-her) [20]. Sessions were delivered by a health coach who had undergone intervention specific training. Intervention content focused on simple, pregnancy specific healthy eating and physical activity messages as well as encouraging healthy GWG according to the IOM guidelines [6] supported by behaviour change strategies designed to optimise lifestyle, GWG (as previously described) and reduce post-partum weight retention in an individualised, interactive environment. Behaviour change and self-management strategies included goal setting, addressing barriers, positive self-talk and relapse prevention in the form of self-directed small, sustainable adjustments to dietary intake and physical activity. Personal goals were discussed and revised at each session, behavioural skills practised and personal action plans supported by written hand-outs. Recommended self-monitoring resources included pedometers and the use of weight gain charts based on international Institute Of Medicine (IOM) recommendations for weight gain during pregnancy [21]. On-going contact and support with mobile phone SMS text messages, personalised by participant name, were provided throughout the study duration commencing from the third lifestyle session. Text messages reinforced simple intervention health messages for diet, physical activity, behaviour change and relapse prevention. Two healthy lifestyle postcards were also sent at 30 and 34 weeks gestation to maintain engagement and remind participants of the simple health messages. Southern Health Research Advisory and Ethics Committee approved the study and all participants gave written consent.

### Measures

Outcome measures were completed at baseline (12–15 weeks), 28 weeks gestation and 6 weeks postpartum. Data collected at 28 week gestation has been previously reported, with postpartum data presented here.

#### Anthropometrics

Anthropometric assessment included weight on an electronic scale measured to the nearest 0.1 kg (Tanita model BWB-800 Digital Scale, Wedderburn Scales, Melbourne, Australia) and height measured by a registered nurse unaware of participant allocation at all time points. Measured weight at baseline was used for all analyses due to inaccuracies associated with self-reported weight [22].

#### Physical activity

The Yamax Digiwalker SW-700 Pedometer (Yamax Corporation, Tokyo, Japan) was used to assess the number of free-living steps per day as a tool with demonstrated accuracy in pregnancy, as we have previously reported [23]. Pedometers were sealed and worn for a minimum of three to seven consecutive days during waking hours, including at least one weekend day, which has previously shown to be reliable for estimating weekly physical activity [24]. All participants were advised on correct usage and provided with a diary to record wearing times. A full day was considered as wearing the device for at least eight daytime hours and a half day was considered as less than eight hours, but more than three hours. If worn for less than three hours a day, this was treated as a missing day. Readings were processed to provide average daily step count according to total days worn.

### Gestational diabetes diagnostic criteria

#### GDM diagnosis

GDM was diagnosed based on the Australasian Diabetes in Pregnancy Society (ADIPS) criteria including the presence of either a fasting venous plasma glucose level of ≥99 mg/dl (≥5.5 mmol/L) and/or a 2 hour level of ≥144 mg/dl (≥8.0 mmol/L) following a 75 g oral glucose tolerance test (OGTT) [25].

#### Statistical analysis

All data are presented as mean ± SD with 95% confidence intervals (CI) unless otherwise stated. Change in variable was defined as the percentage change over time (baseline to 6 weeks postpartum). Two-tailed statistical analysis was performed using SPSS for Windows 20.0 software (SPSS Inc, Chicago, USA) with statistical significance set at α level of p < 0.05. Between group differences were assessed using Univariate analysis (continuous variables) and Chi-Square Tests (categorical variables) at baseline and for outcome data at 6 weeks postpartum. Significant univariate variables at 6 weeks postpartum were entered in to multivariable regression analysis to assess independent predictors of change in weight over time (baseline to 6 weeks postpartum). Regression analysis was also used to assess differences in physical activity (steps/day) at baseline and 6 weeks postpartum between groups.

The sample size for this intervention was calculated based on an a priori between group difference in BMI of 0.8 kg/m^2^ indicated as the average weight retained following a pregnancy in Australian women from the Australian Longitudinal Women’s Health Study [5]. With a 10% attrition rate (informed by previous HeLP-her interventional studies in other populations and settings [20]), 222 women in total were required. The sample size calculation was based on a power of 0.80, a significance level of 5% (2-sided) and a standard deviation of 2.0 kg/m^2^, in line with previous studies [26].

## Results

### Demographics

**Figure 1:**
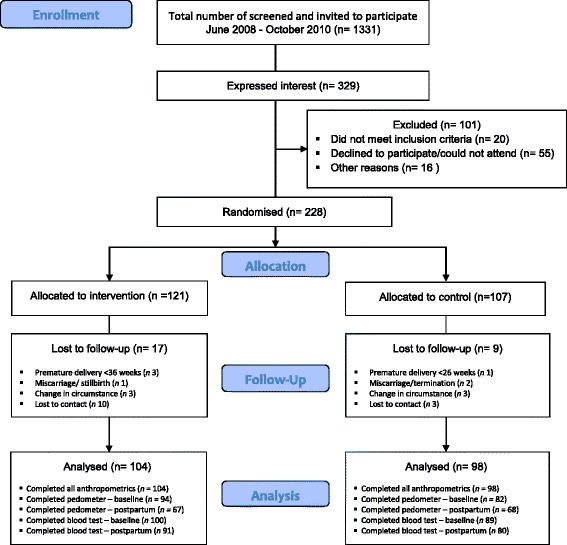
**CONSORT diagram.**

In total, 1331 women were identified at booking visits by midwives as being at risk for GDM using a simple validated screening tool and were invited by an invitation flyer and follow-up phone call to participate in the trial. Of these, 329 expressed interest (25% response rate) and 228 were recruited and randomised with 121 women allocated to intervention and 107 to control at baseline (summarised previously [17]; CONSORT information provided in Figure 1). Mean gestation of women at baseline was 14 ± 0.8 weeks [17]. Of the women allocated to the intervention, 95% attended session two, 89% session three and 93% session four. At 6 weeks postpartum, 104 women from the intervention group and 98 from the control group completed outcome measures, with an overall attrition rate of 11.4%, including lost to follow up and pre-term births defined as ≤36 weeks gestation.

Baseline demographics were similar between intervention (n = 121) and control (n = 107) groups with a mean age of 32.3 ± 4.7 and 31.7 ± 4.4 years, respectively. In recruiting a high-risk cohort, approximately 40% of women were obese with no difference between groups (mean BMI 30.4 ± 5.6 and 30.3 ± 5.9 kg/m^2^ in intervention and control groups, respectively). Women were ethnically diverse with ~35% of women in both groups born in Southern Asia (i.e. India, Pakistan, Bangladesh, Sri Lanka) with a further ~35% Australian born overall. Factors related to socio-economic status, including level of education and total annual household income also did not differ between groups and the four local government areas (LGA) containing the three hospitals were classified as ‘average socio-economic advantage’ based on Australian census data [27]. All baseline demographic characteristics are presented in Table 1.

**Table 1:** **Participant demographic characteristics**

**Variable**	**Control (** ***n*** **107)**	**Intervention (** ***n*** **121)**	**P**
*Demographics*			
Age, yrs	31.7 (4.4)	32.4 (4.7)	0.23
Weight, kg	77.3 (17.7)	78.7 (18.5)	0.58
BMI, kg/m^2^	30.3 (5.9)	30.4 (5.6)	0.49
*Body Mass Index (kg/m* ^*2*^ *) %*			
≤29.99	50.4	45.8	0.57
≥30.00	49.6	54.2	
*Country of Birth %*			
Australia	35.8	38.8	0.77
Southern Asia	36.7	35.8	
Other	27.5	25.4	
*Education %*			
University degree or higher	47.8	50.5	0.40
*Household Income ($) %*			
<40,000	34.8	30.3	0.95
40-60,000	31.5	35.4	
>80,000	20.2	20.2	
*Parity %*			
First pregnancy	40.8	42.3	0.89

BMI (body mass index).

### Impact of the HeLP-her intervention on postpartum weight retention

Overall, weight and BMI change in the control group was 1.96 ± 5.74 kg (0.78 ± 2.26 kg/m^2^) respectively, compared to the intervention group who retained significantly less weight and BMI overall at 0.51 ± 4.48 kg (0.22 ± 1.72 kg/m^2^). The between group difference was 1.45 ± 5.1 kg, (95% CI: −2.86,-0.02; *p* < 0.05).

On unadjusted univariate analysis, intervention group allocation, higher baseline BMI, GDM diagnosis at 28 week, country of birth, higher age, level of educational attainment, and parity were predictors of change in weight over time (baseline to 6 weeks postpartum). When entered in to multiple linear regression, intervention group allocation, higher baseline BMI, GDM diagnosis, country of birth and higher age were independent predictors of lower weight retention at 6 weeks postpartum (Table 2). Independent predictors were explored on univariate analysis below. Additional factors including physical activity levels and breast feeding behaviours did not significantly impact on weight retention (Table 3).

**Table 2:** **Regression analysis for independent predictors of weight change over time (baseline to 6 weeks postpartum)**

**Variable**	**b (95% CI)**	***p***	**B**	***p***
Group	−0.14 (−2.87, −0.02)	0.047	−0.13 (−2.54, −0.03)	0.04
Baseline BMI	−0.33 (−0.41, −.018)	<0.001	−0.18 (−0.29, −0.03)	0.02
Age	−0.30 (−0.49, −0.19)	<0.001	−0.19 (−0.35, −0.07)	<0.01
Diagnosed GDM				
Negative	1			
Positive	−0.34 (−5.69, −2.49)	<0.001	−0.32 (−5.38, −2.33)	<0.001
Country of Birth				
Australia	1			
Asian/Other	0.28 (1.56, 4.41)	<0.001	0.16 (0.00, 3.29)	0.050
Parity				
First pregnancy	1			
One or more previous	−0.25 (−3.98, −1.15)	<0.001	−0.11 (−2.45, 0.23)	0.10
pregnancies				
Level of Education				
Incomplete Education	1			
High school/college or above	0.20 (0.22, 1.27)	<0.01	0.01 (−0.48, 0.54)	0.91

BMI (body mass index); GDM (gestational diabetes mellitus).

**Table 3:** **Outcome variables at 6 weeks postpartum**

**Outcome variable**	**Control (** ***n*** **98)**	**Intervention (** ***n*** **104)**	**P**
*Weight*			
Weight, kg	79.3 (16.8)	79.2 (17.6)	0.73
BMI, kg/m^2^	30.8 (5.6)	30.8 (5.5)	0.99
Weight Change, kg	2.0 (5.7)	0.5 (4.5)	0.047
BMI Change, kg/m^2^	0.8 (2.3)	0.2 (1.7)	0.046
*Birth*			
Mean Gestation, wks	39.2 (1.84)	39.3 (1.68)	0.77
Birth Weight, kg	3.3 (0.5)	3.4 (0.6)	0.11
*Breast Feeding Status (%)*			
Exclusively breast	68.6	63.4	0.59
Breast and Formula	15.7	22.5	
*Physical Activity*			
Steps/day - Baseline	5438 (3145)	5984 (3095)	0.4
Steps/day - Postpartum	5511 (5973)	6245 (4226)	0.6

BMI (body mass index).

### Sub analysis of predictors of weight change

#### Impact of baseline BMI on weight retention

Overall, there was a significantly greater increase in weight over time (baseline to 6 weeks postpartum) in overweight (<30 kg/m^2^) women, compared to obese (>30 kg/m^2^) women (2.42 ± 4.87 vs. -0.51 ± 5.12 kg, *p* < 0.001). There was a between BMI group (overweight vs. obese) difference in weight change of 2.93 ± 4.99 kg (95% CI: 1.5, 4.3; *p* < 0.001). When analysing intervention effects within BMI groups, weight retention postpartum was greater in the overweight control group compared to overweight intervention group (3.33 ± 5.56 vs. 1.48 ± 3.86 kg [95% CI: 1.89, 4.77] *p* < 0.05) with a difference of 1.86 ± 4.71 kg (95% CI: 0.10, 3.59; *p* < 0.05) between groups. Within the obese women, weight returned to baseline with no significant difference between intervention and control groups (−0.76 ± 4.91 vs. -0.22 ± 5.40 kg, respectively, p = 0.63).

#### Impact of GDM diagnosis on weight retention

Overall, there was a significantly greater increase in weight over time (baseline to 6 weeks postpartum) in those without a diagnosis of GDM at 28 weeks compared to those with a diagnosis of GDM (n = 50 overall) (2.17 ± 4.99 kg vs. -1.92 ± 4.47, *p* < 0.001). There was a between GDM group difference in weight change of 4.09 ± 4.73 kg (95% CI: −2.48, −5.69, *p* < 0.001). When analysing intervention effects within GDM groups, weight retention postpartum was significantly reduced in the GDM negative intervention group, compared to the GDM negative control group (1.36 ± 3.87 kg vs. 3.04 ± 5.89 kg, p < 0.05) with a difference of −1.68 ± 4.88 kg (95% CI: −3.25, −0.11; *p* < 0.05) between groups. Within the GDM positive women, there was no significant difference between intervention and control groups (−3.09 ± 5.67 vs. -1.89 ± 3.73, p = 0.45).

#### Impact of country of birth on weight retention

Overall, there was a significantly greater increase in weight over time (baseline to 6 weeks postpartum) in non-Australian born women compared to Australian born women (2.34 ± 4.95 vs. -0.65 ± 5.00 kg, *p* < 0.001). There was a between group (non-Australian vs. Australian) difference in weight change of 2.93 ± 4.99 kg (95% CI: 1.5, 4.3; *p* < 0.001). When analysing intervention effects within groups, weight retention postpartum was significantly reduced in non-Australian born women in the intervention group, compared to the control group by 6 weeks postpartum (1.13 ± 4.11 kg vs. 3.66 ± 5.47 kg, *p* < 0.01) with a between group difference of 2.53 ± 4.79 kg (95% CI: −4.23, −0.84; *p* < 0.01). Within Australian born women, there was no significant difference between intervention and control groups (−0.56 ± 4.93 kg vs. -0.74 ± 5.14 kg, p = 0.87).

#### Impact of age on weight retention

Overall, there was a significantly greater increase in weight over time (baseline to 6 weeks postpartum) in younger women below the cohort mean age (31.9 years) compared to those above the mean age (2.39 ± 5.27 kg vs. -0.41 ± 4.49 kg, p < 0.001). There was no significant intervention effect on weight change in both younger and older women.

## Discussion

Following the delivery of the HeLP-her intervention during pregnancy, we report a reduction in weight retention at 6 weeks postpartum, compared to standard antenatal care. These findings persisted after adjustment for baseline BMI, GDM diagnosis, country of birth and age, which also independently predicted weight retention at 6 weeks postpartum. Exploratory analysis demonstrated a greater intervention effect in overweight compared to obese women, in those not diagnosed with GDM compared to those diagnosed with GDM, in non-Australian born compared to Australian born women, with significant treatment group differences by 6 weeks postpartum.

Longitudinal studies demonstrate rapid weight gain in reproductive aged women, increasing yearly by ~700 grams, equivalent to ~7 kg per decade [5]. Weight retention associated with pregnancy is common, exacerbating background weight gain in younger women [5]. Therefore, the degree of prevention of postpartum weight retention in the current study is of public health significance. Previous findings indicate that natural weight retention at 6 weeks postpartum is ~2.5 kg [28], however varies depending on population, time course following pregnancy and accuracy of measurement. Our current results are consistent with this degree of weight retention in our control group highlighting pregnancy as a key high risk life stage for targeted intervention. In the years following pregnancy, women retain a self-reported 2-3 kg based on reports from the Australian Longitudinal Study on Women’s Health (ALSWH) study [29], with similar or higher findings reported in the US [21]. Increased weight retention from excess GWG predicts long term obesity with a 3 fold increased risk of being overweight 16 years post-pregnancy if GWG exceeds IOM guidelines, as well as increasing comorbidities including central adiposity and hypertension [30]. Data from the Nurses’ Health Study data suggest that long term health risks including the risk of coronary heart disease increases by 3.1% for each kilogram in weight gained [31] and the risk of diabetes increases linearly from a body mass index of 22 kg/m^2^ [32]. For these reasons, modest improvements in weight gain prevention are significant from a public health perspective and may impact on longer term weight, obesity rates and potentially on long term complications especially if modifiable health behaviours are sustained. However, longer term follow-up studies are needed to clarify the latter.

With up to 50% of women in developed countries entering pregnancy overweight or obese [33], early preconception education is ideal, yet barriers include engagement, health provider contact and unplanned conception. Engaging women following pregnancy is equally difficult [12]. However, optimising GWG has potential to prevent pregnancy complications, prevent weight retention postpartum and reduce long term weight related complications, yet there is a significant research gap in low-intensity, effective interventions during pregnancy with few extending to, or reporting, postpartum weight retention [12]. Of the limited studies that have, efficacy has not been demonstrated in overweight women, despite frequent contact [34-36] and additional support for excessive gainers [34], with increased intensity potentially contributing to high reported attrition rates of >30% overall across studies [34,35]. Here, we advance knowledge in this field by targeting a group of high risk overweight and obese women in early pregnancy, implementing a non-intensive individualised behavioural change intervention, achieving high retention rates (~90%) and demonstrating significant reduced postpartum weight retention in overweight women. By aligning the intervention with routine antenatal care, we demonstrate opportunity for implementation in to routine antenatal care as an approach to optimise GWG and potentially, improve clinical outcomes.

Consistent with previous research [37], we have shown here that postpartum weight retention is inversely related to pre-regnancy BMI, with non-obese women at greatest risk. It is notable that the current intervention was most effective in this non-obese cohort of women who are at highest risk for weight gain in pregnancy. Further research in obese cohorts is needed with previous research of different intensities producing contradicting effects on weight change in obese women [16,34,36,38,39] and current ongoing research with larger populations in the US and Europe may provide additional insight. As noted previously [40], the intervention was more effective in reducing postpartum weight retention in non-Australian born women, comprising ~60% of the study population. The prevention of weight gain reported (~2.5 kg between treatment groups) is important given the greater risk for weight gain in pregnancy and for GDM diagnosis, which may, in part relate to cultural beliefs and practices, including reduced engagement in physical activity [33,40,41]. A diagnosis of GDM at 28 weeks gestation was associated with reduced weight retention overall, most likely contributed to by increased health professional engagement and intensive medical intervention following GDM diagnosis and potentially reducing intervention effect in this sub-group.

Interestingly, in the current study we were unable to demonstrate significant changes or between group differences in physical activity as measured by daily step count. This could be explained by the self-management intervention design, with small, sustainable adjustments to a range of healthy lifestyle behaviours encouraged in an individualised environment according to participant goals, which were variable between intervention participants. Strengths of the current study include an intervention that is based on an established behavioural theory and informed by our previous lifestyle interventions programs that have been effective in other populations and settings [20]. The low-intensity study design is feasible and sustainable, reflected by factors including delivery by a trained health coach, a low attrition rate of ~10%, primarily attributed to pre-term birth and miscarriage and a high compliance rate of between 90-95% of allocated intervention participants receiving the intended intervention content. Further strengths include a pre-specified population of high-risk women identified at increased risk of GDM on a validated screening tool [7], prospective anthropometric assessment by investigators blinded to group allocation and successful delivery to an ethnically diverse, heterogeneous group of women of moderate socio-economic advantage, indicating that the messages implemented were easily transferable across sociocultural beliefs and practices.

## Conclusion

Results demonstrate an evidence-based, low-intensity, self-management intervention delivered in early pregnancy reduces postpartum weight retention in high-risk overweight, culturally diverse women, who currently comprise the highest risk of weight gain in general and obstetric populations. With effective engagement, motivation and adherence, further translational research is now required to enable integration and broad scale up into routine antenatal care. This needs to include health economic evaluation, production of accessible resources, cultural change and education of health professionals, engagement of policy makers and funders and changes in health care delivery systems to enable population based prevention of postpartum weight retention.

## Ethics approval

The Southern Health Research Advisory and Ethics Committee approved the study and all participants gave written informed consent. Approval date 1/4/2008; project number 07216C. Clinical Trial Registration: Australian New Zealand Clinical Trial Registry Number: ACTRN12608000233325. Registered 7/5/2008.
